# Somatic versus Dendritic Resonance: Differential Filtering of Inputs through Non-Uniform Distributions of Active Conductances

**DOI:** 10.1371/journal.pone.0078908

**Published:** 2013-11-05

**Authors:** Ekaterina Zhuchkova, Michiel W. H. Remme, Susanne Schreiber

**Affiliations:** Institute for Theoretical Biology, Humboldt-Universität zu Berlin, Berlin, Germany; McGill University, Canada

## Abstract

Synaptic inputs to neurons are processed in a frequency-dependent manner, with either low-pass or resonant response characteristics. These types of filtering play a key role in the frequency-specific information flow in neuronal networks. While the generation of resonance by specific ionic conductances is well investigated, less attention has been paid to the spatial distribution of the resonance-generating conductances across a neuron. In pyramidal neurons – one of the major excitatory cell-types in the mammalian brain – a steep gradient of resonance-generating h-conductances with a 60-fold increase towards distal dendrites has been demonstrated experimentally. Because the dendritic trees of these cells are large, spatial compartmentalization of resonant properties can be expected. Here, we use mathematical descriptions of spatially extended neurons to investigate the consequences of such a distal, dendritic localization of h-conductances for signal processing. While neurons with short dendrites do not exhibit a pronounced compartmentalization of resonance, i.e. the filter properties of dendrites and soma are similar, we find that neurons with longer dendrites (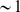 space constant) can show distinct filtering of dendritic and somatic inputs due to electrotonic segregation. Moreover, we show that for such neurons, experimental classification as resonant versus nonresonant can be misleading when based on somatic recordings, because for these morphologies a dendritic resonance could easily be undetectable when using somatic input. Nevertheless, noise-driven membrane-potential oscillations caused by dendritic resonance can propagate to the soma where they can be recorded, hence contrasting with the low-pass filtering at the soma. We conclude that non-uniform distributions of active conductances can underlie differential filtering of synaptic input in neurons with spatially extended dendrites, like pyramidal neurons, bearing relevance for the localization-dependent targeting of synaptic input pathways to these cells.

## Introduction

Responses to synaptic input are shaped by a neuron's membrane properties. In the subthreshold membrane potential range such filtering can have low-pass or resonant characteristics – i.e., a cell either shows the largest amplitude responses to low input frequencies, or it responds maximally to input in a particular frequency band (see [1] and references therein). Such resonant properties of neuronal membranes are thought to play an essential role in the generation of brain rhythms associated with various behavioral and perceptual states [2]. Membrane-potential resonances are generated by voltage-dependent conductances that actively oppose changes in membrane potential and activate slowly compared to the membrane time constant [1]. A key player in the generation of subthreshold resonance is the h-type current, which is carried by the hyperpolarization-activated, cyclic nucleotide-gated HCN channels (h-channels). Its voltage-dependent dynamics underlie membrane-potential resonance in, e.g., cortical and hippocampal pyramidal cells [3]–[9]. In the hippocampus it is thought to play a central role in the generation of local-field theta oscillations (4–12 Hz range; [10], [11]).

While a *somatic* subthreshold resonance can be well described by a single compartment neuron model [12]–[14], h-channels are, in fact, distributed in a highly non-uniform way across the soma and dendrites in various types of neurons [15]. In particular, pyramidal cells have dendritic trees of considerable spatial extent and show a steep gradient of h-conductances along the dendrite. Experimental work demonstrated that the density of h-channels increases up to 60-fold with somatic distance along the apical dendrites of pyramidal cells in hippocampus and neocortex [15]–[19]. An important consequence of such location-specific channel expression is that the characteristics of the membrane-potential resonance typically also vary across the neuron [20], [21], and may hence be expected to affect the processing of synaptic input in a location-dependent manner.

Here, we aim to understand how a distal, dendritic concentration of resonance-generating conductances affects the response to dendritic versus somatic input. Using an analytically tractable neuron model, we show that a predominant expression of resonance-generating channels in distal dendrites can be responsible for a strong dendritic resonance that shapes the somatic response to dendritic input, without affecting the response to somatic input. A key requirement is that the resonant conductances are concentrated approximately one electrotonic space constant (or more) away from the soma, a condition that seems particularly applicable to the extended apical, dendritic trees of pyramidal neurons (see, e.g., [22], [23]). An important consequence of a dendritic localization of resonant conductances is that experimental classification of resonant versus nonresonant cells may be misleading when based on somatic recordings. Finally, we demonstrate that dendritically-generated membrane-potential oscillations (MPOs) may still propagate to the soma where they can be picked up by somatic measurements while the dendritic resonance itself is not reflected in somatic input-response characteristics.

## Results

**Figure 1:**
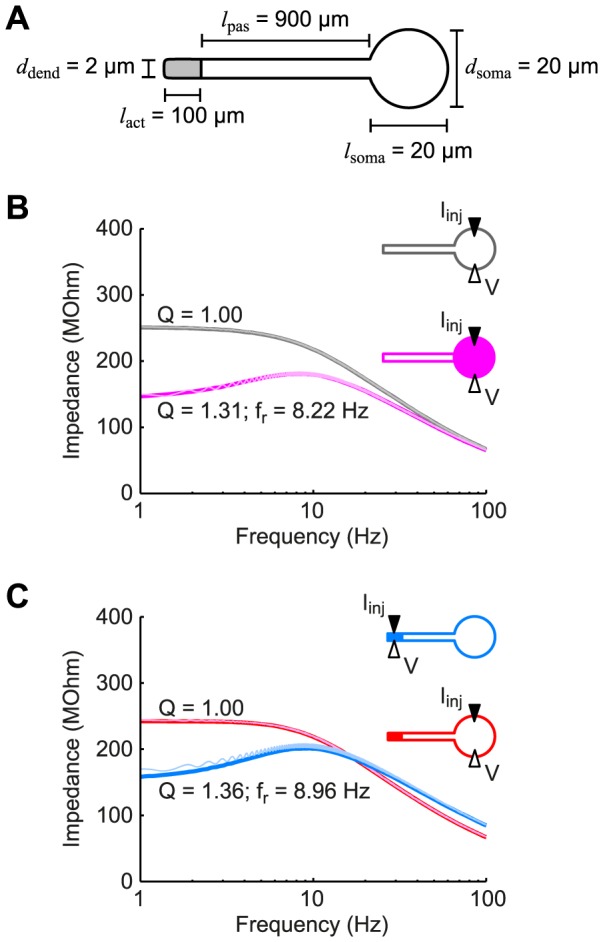
Dendritic resonance may not be detectable in somatic measurements. (A) Schematic of the standard model used throughout the study showing the spatial dimensions of the soma and dendrite. The soma and proximal part of the dendrite had passive membrane properties while the distal, dendritic end (gray) had voltage-dependent h-conductances. (B) The somatic input impedance of a passive neuron (gray) demonstrated low-pass behavior. When h-conductances were added to the soma (magenta curve), this resulted in a band-pass filtered response. (C) If the h-conductances were located in the distal dendritic end as in panel (A), a resonance was observed in the dendritic input impedance (blue curve), but was not detectable somatically (red curve). Thin curves in panels (B) and (C) correspond to numerical results from the full nonlinear model (see Methods).

In this study, we investigated the consequences of a distal, dendritic expression of resonance-generating h-channels for neuronal signal processing. We focused on how such a channel localization affects the neuronal response to dendritic and somatic input. Concomitantly, we considered the experimental detectability of subthreshold resonance in somatic measurements of such neurons. To quantify the effects of non-uniform h-channel distributions on input filtering and detectability of resonance, we derived a minimal mathematical model of a spatially extended neuron with active channel dynamics (for details see Methods). The model consisted of a soma with a finite dendritic cable, similar to the Rall model of the motoneuron [24]. We extended the dendritic cable with a lumped, active compartment representing the distal dendrites expressing the h-channels (Figure 1A). This captured the steep asymmetry of h-channel density along the apical dendrite of pyramidal neurons, while allowing us to treat the model analytically. Spatial dimensions of our reference model were in accordance with morphological data on cortical pyramidal cells [25]. The description of the h-current, 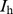, was based on recordings from cortical neurons [26] and consisted of a dominant fast component (40 ms time constant) and a smaller slow component (300 ms). The biophysical properties of h-channels can give rise to a resonance within the theta range (see, for example, [7], [27]–[29]). To allow for mathematical analysis of the frequency-dependent input filtering of the neuron model, we linearized the h-current around a holding potential 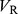 (here, 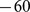 mV; see Methods and [30]).

### Visibility of dendritic resonance in the somatic compartment

The subthreshold voltage response to current input can be characterized by the input impedance, which is quantified based on current injection in one site and recording of the voltage response at the same site. Mathematically, it is a complex-valued function of the input frequency, defined by the ratio of the voltage to the input current (see Methods). Although it is measured by current injection at one site and recording the voltage response at that same site, it is important to realize that the input impedance is not only determined by local membrane properties, but is also shaped by membrane properties of other, neighboring compartments depending on the electrical coupling with those compartments. Before considering a distal concentration of h-channels, we first illustrate two standard cases comprising an entirely passive model neuron and a model neuron with an exclusively somatic expression of h-channels (Figure 1B). We considered the somatic input impedance (i.e., both injection of current and measurement of voltage at the soma). As one would expect, the entirely passive model showed a low-pass somatic input impedance (Figure 1B, gray curve), whereas the model with somatic h-conductances showed a resonance in the somatic input impedance (Figure 1B, magenta curve). The resonant (or band-pass) filter peaked at a frequency of 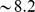 Hz. We characterized the “quality” of the resonance with the so-called “Q-value”: the ratio of the impedance amplitude at the resonant frequency to the input resistance (see [31], [32]), which yielded a value of 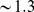 for this model. Note that while Q-values larger than 1 denote a band-pass filter, in experimental studies one should generally rely on larger Q-values (usually more than 1.2) for identification of membrane resonances in order to surpass the intrinsic noise level [13].

The resonance observed above was clearly reflected in the input impedance, as the latter was determined in the compartment where the resonant h-conductances were located. Next, we turned to the model described in Figure 1A with h-channels distributed in a pyramidal-cell like manner (i.e., concentrated in the distal part of the dendrite). Again, when we measured the input impedance in the active compartment (here, current injection and response measurement at the distal dendritic end) we observed a strong resonance (Figure 1C, blue curve, Q = 1.36). However, when we determined the input impedance at the soma, we observed a low-pass filter, as if there was no resonant current present in the neuron (Figure 1C, red curve, Q = 1.00). Note that the analytically calculated impedance profiles (Figure 1B–C, thick curves) coincided with numerical simulations of the response of the full nonlinear model (thin curves; see Methods), showing that the nonlinear models were very well approximated by the analytically treatable linear ones.

The natural question to ask next is whether the resonant dendritic responses are, in fact, visible at the soma. For this we characterized the signal filtering along the dendritic cable using the so-called transfer impedance (see Methods). Whereas the input impedances that we computed above characterized the voltage response at the same location where the current input was provided, transfer impedances relate the current injected in one location to the voltage response that this current elicits in a different location. Though the transfer impedance is symmetrical in the sense that it is identical in the opposite direction (i.e., when switching the input and recording sites; see, e.g., [33]), we typically refer to the transfer impedance from dendrite to soma, since this is the usual direction of input-output flow in a neuron.

**Figure 2:**
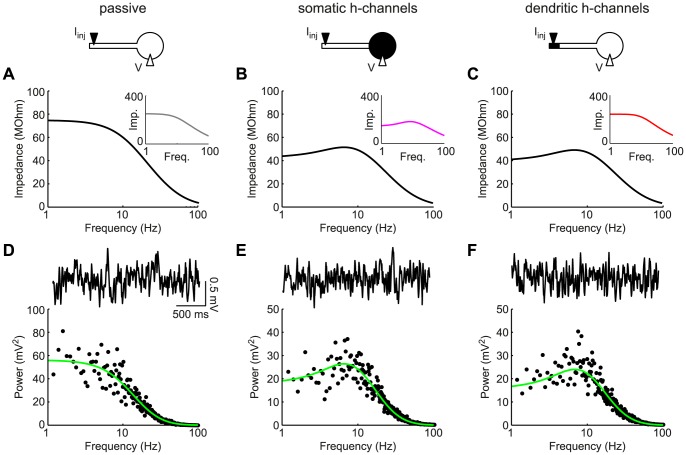
Signals of dendritic origin reflect dendritic resonance in the somatic compartment. (A–C): Dendro-somatic transfer impedances for an entirely passive neuron (A), a cell with h-channels localized in the soma (B), and a neuron with h-channels in the distal end of the dendrite (C). Insets show local somatic impedances from Figure 1. In contrast to the input impedances, transfer impedances showed a resonant peak, independent of whether h-channels were located somatically (B) or dendritically (C). (D–F): For all three models white noise current was injected at the distal dendritic end. Based on numerical simulations of the (in the two cases with h-conductances nonlinear) model equations, example voltage traces were obtained (solid black curves). Power spectra of responses (black dots) agreed with the squared dendro-somatic transfer impedances (green curves). Dendritic resonance-induced MPOs could be seen in somatic measurements despite the apparent absence of the resonance (F).

We considered the same three neuron models as in Figure 1. As expected, the transfer impedance of the passive model showed low-pass characteristics (Figure 2A; the inset depicts the somatic input impedance from Figure 1A). The model with somatic h-channels (Figure 2B) not only showed a resonant peak in the somatic input impedance (inset), but also in the transfer impedance (black curve). Hence, the somatic response is qualitatively the same for somatic input as for dendritic input. However, in the model with h-channels in the distal dendritic end, the transfer impedance and the somatic input impedance are qualitatively different (Figure 2C): whereas the somatic response to somatic input demonstrated low-pass characteristics (inset), the somatic response to dendritic input showed a resonance (black curve). Hence, the two ‘pathways’ were differentially filtered. Importantly, this also illustrates that for a neuron with a pyramidal-cell-like distribution of h-conductances, a somatic assessment of the input impedance could misleadingly suggest that the neuron cannot show band-pass filtering, whereas in fact it can for dendritic input.

Note that the models with somatic or distal dendritic h-conductances showed almost identical resonant transfer impedance profiles (Q-values of 1.25 and 1.28 and resonant frequencies of 6.58 Hz and 6.84 Hz, respectively). This is in accordance with results from [34], who demonstrated that the transfer impedance is hardly affected by the precise distribution of 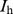 between input and output locations as long as the total h-conductance remains the same, which was indeed the case for the two models above.

### Resonance-associated membrane-potential oscillations

Resonant membrane properties can underlie the generation of membrane-potential oscillations (MPOs) through an interplay between the resonant conductances and noise (from ion channel stochasticity or other sources; [13], [35], [36]). Intuitively, cell-intrinsic or synaptic broadband noise is filtered by the subthreshold resonance, resulting in noise-driven voltage fluctuations whose preferred frequency is reflected in a peak (at non-zero frequency) in their voltage power spectrum. MPOs have been demonstrated in various cell types, including stellate cells from entorhinal cortex [13], [37] as well as pyramidal cells and interneurons from hippocampus [38], [39]. Though resonance and noise-driven MPOs can be considered two sides of the same coin, these phenomena can also occur independently. Resonance need not be accompanied by MPOs if the noise amplitude is small. However, the opposite case, MPOs without resonance, is more difficult to explain. A mechanism for the latter case was provided by Richardson and colleagues [40] in a single-compartment model in a narrow parameter range. Here, we show that the spatial separation of h-channels from the soma in a pyramidal-cell-like morphology provides an additional mechanism how MPOs can occur in the apparent absence of somatic resonance.

The results in the previous section suggest that neurons with a distal, dendritic localization of h-conductances could be classified as nonresonant by somatic input impedance measurements, but that band-pass-filtered responses may propagate from the dendrites to the soma. Hence, if the noise source underlying MPOs is located in the distal dendrites, MPOs can be created locally in the distal dendrite and then propagate to the soma. To demonstrate this, we provided white noise current input (representing synaptic or channel noise) to the distal dendritic segment of the three models (passive, somatic h-channels, dendritic h-channels) and measured the voltage response at the soma. From the voltage traces themselves it was not clear whether the somatic response showed any oscillatory components (Figure 2D–F, top traces; see also [35]). However, the voltage power spectra demonstrated maxima in the theta range (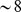 Hz) for both neurons with h-channels, while the passive neuron did not show a preferred frequency. The spectra of the nonlinear models were well-approximated by the squared transfer impedances (multiplied by the noise power spectrum) calculated analytically for the linear models (Figure 2D–F, black dots versus green curves). In summary, somatic MPOs were present even in the absence of somatically-detectable resonance. In general, the extent to which dendritic resonance and MPOs are reflected in the somatic compartment depends on properties of the membrane as well as neuronal morphology. Both aspects are investigated in the following sections.

### Conditions for differential filtering of somatic and dendritic inputs

**Figure 3:**
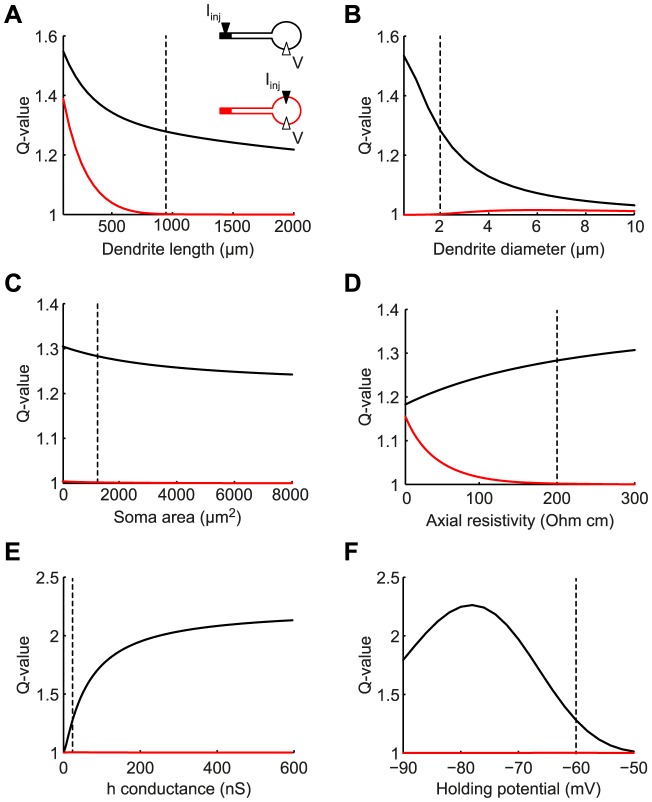
Dendritic resonance can be reflected somatically despite low-pass filtering of somatic inputs. Q-values of somatic input impedance (red curves) and dendro-somatic transfer impedance (black curves) when varying dendrite length (A), dendrite diameter (B), soma surface area (C), axial resistivity (D), h-conductance (E), and membrane holding potential (F). Vertical dashed lines indicate the default parameter values used in this study. There were large regions in the parameter space where MPOs propagated to the soma, but resonance was not detectable somatically.

To identify the conditions when dendritic resonance does not affect somatic input, while maintaining an effect on dendritic signals reaching the soma (such as synaptic inputs or MPOs), we analyzed the transfer and input impedances in the model with distal dendritic h-channels across a range of morphological and electrical parameters (Figure 3). Such regimes are defined by a dendro-somatic transfer impedance exhibiting a substantial peak (at non-zero frequency) while the somatic input impedance is low-pass. Quantitatively, we compared the Q-value of the somatic input impedance (red lines) with the Q-value of the dendro-somatic transfer impedance (black lines) when varying the model parameters.

For the dendritic compartment with resonant conductances not to affect the response of the somatic compartment to somatic input, the two compartments need to be electrotonically sufficiently distant from each other. Hence, model parameters of interest can be predicted from classical cable theory (see, e.g., [41]). The electrotonic distance between two points along a cable increases with the physical distance and the axial resistivity, while it decreases with the cable diameter and the membrane resistance. Furthermore, the larger the (passive) soma membrane area, the less its response is affected by dendritic membrane properties.

Indeed, results from our model agree with those predictions. A key parameter determining the presence of a resonance in the somatic input impedance was the length of the passive dendrite (Figure 3A). Long dendrites displayed a low-pass somatic input impedance (Q-value for red curve is 1), while the transfer impedance showed a strong resonance over the entire depicted range. Hence, when the h-conductances were electrotonically remote from the soma (here, larger than 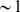 space constant, which was 527 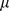
m), their effects were not detectable in the somatic input impedance.

In our default parameter set (indicated by vertical dashed lines in all panels of Figure 3), the somatic input impedance did not show a resonance. This changed only slightly when increasing the dendrite diameter (Figure 3B). While this decreased the electrotonic segregation between soma and the distal dendritic compartment, it also decreased the contribution of the active, distal compartment to the model's response, hence strongly decreasing the Q-value of the transfer impedance. In contrast, only a small decrease of the transfer impedance was observed when we increased the soma surface area (Figure 3C), while the somatic input impedance was unaffected.

Another important parameter controlling the resonance of the somatic input impedance was the axial resistivity, 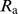. Decreasing this parameter revealed the dendritic resonance in the soma (Figure 3D), because it decreased the electrotonic separation between the active dendritic segment and the soma. It is important to note here that experimental estimates of 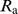 vary considerably, both for the same type of neuron as well as between different types of neurons. For example, a recent study found that the axial resistivity of CA1 pyramidal neurons lies within the range of 139–218 Ohm cm [42], which is approximately twice as high as what was reported for cortical layer V pyramidal neurons (70–100 Ohm cm) [43].

Parameters that affected the resonant membrane properties directly, such as the h-conductance in the distal dendritic end (Figure 3E) and the cell's holding potential (Figure 3F), also controlled the Q-values of the transfer impedance. However, they did not change the low-pass nature of the somatic input impedance, since these parameters did not affect the electrotonic separation between the soma and the active, distal dendritic segment.

**Figure 4:**
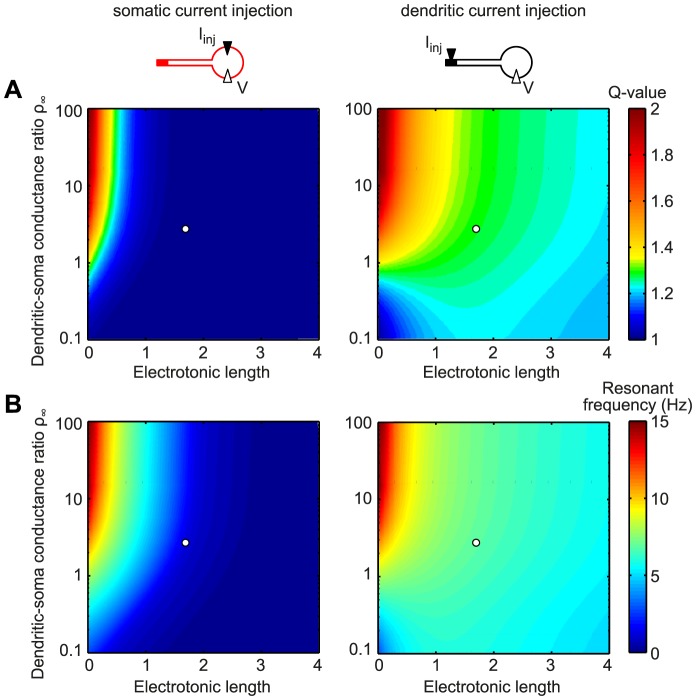
For electrotonically large cells the origin of input determines the “visibility” of dendritic resonance in somatic measurements. Color-coded Q-value (A) and resonant frequency (B) of the somatic input impedance (left panels) and the dendro-somatic transfer impedance (right panels). The somatic input impedance exhibited a resonance only for short electrotonic lengths and large dendritic-soma conductance ratios 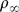. In contrast, the transfer impedance showed a resonance across the whole parameter range with the exception of cells that are electrotonically small and have a low dendritic-soma conductance ratio. White circles correspond to the default model with electrotonic length 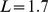 and conductance ratio 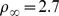.

As an alternative to varying the physiological parameters that determine cell morphology (dendrite length and diameter, and soma surface area), we also systematically analyzed the electrotonic properties of the cell: the electrotonic length of the passive stretch of dendrite that connects to the active distal compartment as well as the ratio of the dendritic input conductance to the soma conductance, 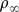 [24], [41]. The dendritic-soma conductance ratio 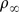 indicates the relative electrical “magnitude” of the two elements; for a given dendrite, 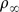 is inversely proportional to the size of the soma. For most of the parameter range that we explored, the somatic input impedance did not show resonance (dark blue color in Figure 4A, left panel; white circle denotes default model parameters) while the transfer impedance (right panel) did, with resonance frequencies between 5 and 10 Hz (Figure 4B). Only electrotonically small neurons (electrotonic length below 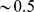) with large 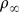 (i.e. small soma, 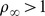) displayed resonances for somatic input.

### Analysis of different dendritic morphologies and conductance distributions

**Figure 5:**
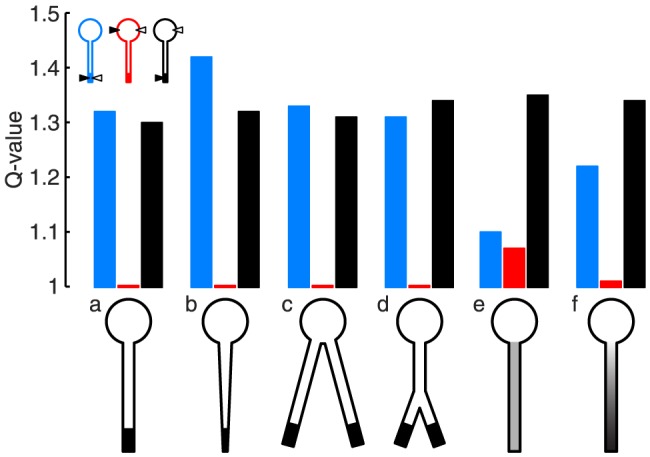
Differential filtering of somatic and dendritic input persists for a variety of cell morphologies and a gradual distribution of the active current. Q-values of the dendritic input impedance (blue), somatic input impedance (red), and dendro-somatic transfer impedance (black). *a*: The default model from Figures 1B, 2C, 3, and 4 (length of passive cable 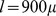
m, length of active distal end 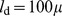
m, dendrite diameter 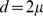
m, peak conductance in active segment 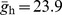 nS). *b*: Tapering of diameter towards the dendritic end (dendrite diameter gradually decreases from 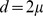
m to 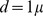
m). *c*: Neuron with two dendrites, both with the same parameters as model *a*. *d*: Branching neuron (length of primary dendrite 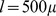
m, length of passive parts of both daughter dendrites 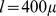
m, length of active dendritic ends 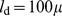
m). *e*: Uniform distribution of h-conductances (peak conductance density 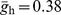 mS/cm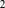). *f*: Exponential distribution of h-conductances (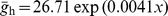 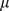
S/cm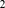). Note that the total h-conductance and the length of the path between distal dendritic end and the soma were the same in all considered cases.

In the previous section we considered the default model neuron with ball-and-stick morphology and a distal dendritic localization of h-channels. As we show next, our results also held for various simple neuronal morphologies and different distributions of h-channels across the dendrite. Besides for our standard model (Figure 5, model *a*), we determined response characteristics for three additional neuron models with distinct morphologies: a cell with a tapering dendrite (model *b*), a neuron with two dendrites (model *c*), and a neuron with a branching dendrite (model *d*). Furthermore, we considered two models with modified spatial distributions of h-channels: a cell with a uniform dendritic distribution (model *e*) and a cell with an exponentially increasing h-channel density that was constrained by experimental data from pyramidal neurons (model *f*) [16], [19]. In all cases, Q-values for the dendritic input impedance, somatic input impedance, and the dendro-somatic transfer impedance were determined.

The resonance of the transfer impedance (red bars) had a similar Q-value (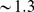) for all six models. This was expected since the models had the same total “number of h-channels” distributed between input and output locations [34]. In contrast, the resonance was absent for the somatic input impedance (red bars) in all models; the only exception was the model with the uniform h-channel distribution (model *e*), which had a similar (local) resonance strength for somatic and for dendritic input, since there was no electrical separation between the soma and the active dendrite. Similarly, local resonance strength for dendritic input was high for all models, except again for model *e*, where resonance was limited because of the lower h-channel density. Note that the model with the experimentally motivated exponential increase in h-channel density along the dendrite (model *f*) gave similar results as the default model *a*; Q-values for the dendritic and somatic input impedances as well as the transfer impedance were approximately the same.

In summary, differential somatic responses to somatic versus dendritic input – and consequently the existence of somatic MPOs in the absence of somatic resonance – are common features of neurons with various morphologies, provided that the majority of resonance-generating conductances is electrotonically sufficiently distant (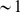 space constant or more) from the soma.

## Discussion

We have used mathematical descriptions of spatially extended neurons to analyze the effect of non-uniform distributions of resonant conductances on the processing of dendritic and somatic input. This was motivated by the finding that h-channels, which underlie subthreshold resonance in many types of neurons, are often – notably in cortical and hippocampal pyramidal neurons – distributed in a highly non-uniform fashion across the soma and dendrites, with most of the channels concentrated in the distal dendrites [15]–[19]. Our results demonstrate that a dendritic resonance may strongly shape the somatic response to dendritic inputs, without affecting the response to somatic input, or indeed, without being detectable with somatic current clamp recordings, as long as the majority of resonant conductances are located in the dendrites, approximately one space constant or further from the soma. In contrast, membrane potential oscillations (MPOs) caused or supported by dendritic resonance can still be discernible in somatic recordings.

Previous mathematical work on input integration in cells with active dendritic currents has typically considered uniform membrane properties, using the so-called quasi-active description of dendritic cables [31], [44]–[46]. In the present study we analytically quantified the effects of a strongly polarized distribution of active conductances on the response to dendritic and somatic input (part of these results are also presented in [47]). To this end, we extended the Rall model of a passive dendritic neuron [24] by including a lumped distal dendritic segment with active membrane properties. We found that the extent to which the distal dendritic resonance affects the somatic response to somatic input depends on the electrotonic separation of the resonant dendritic segments from the soma. If the resonant membrane is close to the soma and the dendritic-soma conductance ratio is large (i.e. the soma is small), the somatic input will activate the resonant current, which, in turn, will shape the somatic response. However, if the resonant membrane is more distant, the somatic input will perhaps propagate sufficiently into the dendrites to activate the distal resonant conductances, but this dendritically filtered response will be further attenuated on the way back to the soma, thereby rendering its effect on the somatic response negligible. In other words, for somatic input to be affected by a distal dendritic resonance and be picked up in a somatic voltage recording, it has to cover the distance between the soma and the resonant membrane twice. In contrast, distal dendritic input will be locally filtered by the resonant conductances, and the response will still be detectable at the soma (unless the electrotonic distance becomes too large). In this case, the distance between the soma and the resonant membrane needs to be covered only once. We demonstrated that these results hold for a variety of cell morphologies and distributions of the resonant conductances.

### Frequency- and location-specific filtering of inputs

The spatial distribution of resonance-generating conductances as analyzed in this study has important implications for neuronal input processing. A high density of h-channels in the distal part of the dendrite will lead to band-pass filtering of synaptic inputs impinging on this part of the dendritic tree, which will also be reflected somatically. Our work shows, however, that somatic input to the same cell may give rise to low-pass responses. This occurs when the dendritic resonance is electrotonically distant and therefore does not substantially affect input to the somatic compartment. Our analysis demonstrates that this requires an electrotonic distance of 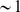 space constant. This argues against such differential filtering to occur in electrotonically compact cells such as cerebellar Purkinje cells [48]. However, the large apical dendritic trees of cortical and hippocampal pyramidal neurons appear particularly suited to allow for the location-specific filtering of inputs (see, e.g., [22], [23]). This is particularly relevant because input projections to neurons often target specific domains of the neuron. For example, in CA1 pyramidal cells, inputs from entorhinal cortex project to the distal dendrites, while inputs from hippocampal CA3 cells arrive proximally to the soma. The steep gradient of h-channels along the dendrites of CA1 pyramidal cells suggests that inputs arriving from these two pathways are subject to distinct filtering (see also [20]). Moreover, spike initiation may be subject to an additional frequency-dependent filter process due to local resonant currents that activate at more depolarized levels [21], [49], [50].

### Membrane-potential oscillations without resonance

Membrane-potential oscillations have been observed in many neuron types, including cells in the entorhinal cortex and hippocampus [13], [37]–[39]. Modeling work suggests that MPOs can result from the interplay between resonance-generating active conductances and noise that arises from, e.g., ion-channel stochasticity [13], [35], [36]. These MPOs are irregular, but their voltage power spectrum exhibits a prominent peak. Usually, it is assumed that if subthreshold MPOs can be detected, also a subthreshold membrane-potential resonance should be present. Our results show, however, that depending on the spatial location of the resonating mechanism, MPOs can be picked up by somatic recordings in the absence of a resonance in the somatic input impedance. This is the case if the resonant conductances are located in electrotonically distant compartments, like the distal ends of apical dendrites, such that the dendritic resonance is not reflected in the local voltage responses to currents injected somatically. Noise-driven MPOs of distal dendritic origin (caused either by channel or synaptic noise) may, nevertheless, still reach the somatic compartment and result in a peaked voltage power spectrum. For completeness, it should be mentioned that an alternative mechanism by which somatic MPOs can occur in the apparent absence of somatic membrane-potential resonance has been reported previously [40]. This single-compartment mechanism does not require a spatial separation of resonance and appears in a narrow parameter regime, where damped oscillations can occur in the absence of membrane-potential resonance.

### Consequences for *in vivo* modulation of neural dynamics

Resonant properties can be dynamically modified via neuromodulation (e.g., changes in acetylcholine levels [51]) or through a variation in the conductance of the membrane (e.g., *in vivo* changes in synaptic input levels [52]). It is tempting to conclude that, hence, also the frequency-dependent filtering of inputs to affected neurons must be changed. However, our results imply that the effect on the frequency-dependent information flow in local circuits depends on the neuronal localization of the modulation. Along these lines, a high-conductance state of the soma may eradicate local somatic resonance properties and detectability of resonance in somatic measurements [52]. Nevertheless, inputs to distal dendritic parts may still be filtered by a dendritic resonance and hence may preferentially contribute to spiking in the resonant frequency range.

### Conclusions

Experimental investigations of the location dependence of input filtering are demanding, as they require recordings (potentially with multiple electrodes) from dendrites, which typically have diameters of less than 1 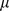
m. Our theoretical study based on simplified morphologies helps to assess the effects of spatially confined resonances. Our results propose that spatial compartmentalization of resonance via non-uniform ion channel distributions could contribute to frequency-dependent information routing in the brain. Accordingly, also the functional effect of neuromodulation and changes in conductance states have to be interpreted with respect to the localization of their action. In particular, for cells with an extended dendritic tree, like cortical or hippocampal pyramidal neurons, it is likely not sufficient to assess fundamental properties of neuronal input processing based on somatic recordings alone.

## Methods

### Mathematical model of a dendritic neuron with a non-uniform distribution of active currents

To mathematically analyze the frequency-dependent response to dendritic and somatic input in a neuron with a strongly polarized distribution of active conductances, we extended the Rall model of a passive dendritic neuron [24] by including an active distal dendritic segment (see Figure 1A). The passive cable equation satisfies
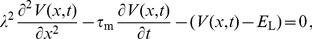
(1)where 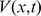 is the membrane voltage along the cable, 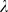 is the space constant, 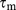 is the membrane time constant, and 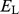 is the leak reversal potential. The soma, represented by a single isopotential compartment, is attached at 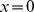:
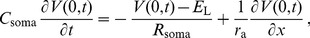
(2)where 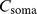 is the capacitance and 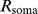 is the membrane resistance of the somatic compartment, and 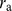 is the axial resistance of the dendritic cable. Our standard model considers that the active conductances are concentrated in a lumped compartment at the distal end of the passive dendritic cable (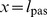):
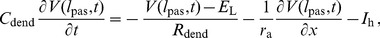
(3)where 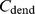 is the capacitance and 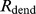 the membrane resistance of the dendritic compartment, and 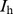 is the voltage-dependent h-current. The model of the h-current is based on [26] (see also [53] and [32]) and consists of a fast and a slow component:
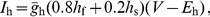
(4)

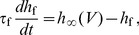
(5)

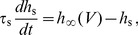
(6)with activation function 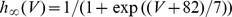, peak conductance 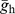, reversal potential 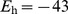 mV, fast activation time constant 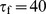 ms, and slow activation time constant 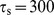 ms.

To allow us to compute the filtering characteristics of the above nonlinear model, we linearized the h-current around holding voltage 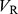. Such a linear approximation retains the activation dynamics of a voltage-dependent current, but loses the nonlinearity of the activation function and the voltage-dependence of the driving force and the activation time constant. The approximation is valid for small voltage changes, however, these voltage changes can, in fact, be quite large (say, 10–20 mV), depending on the specific active current and the type of stimulus. The linearized membrane dynamics of the distal active compartment can now be described as an LRC electric circuit consisting of two phenomenological inductances, three resistances and a capacitance; for perturbations around 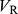 the h-current responds as if the total membrane resistance is in parallel with two inductive branches:
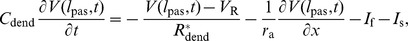


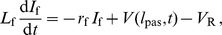


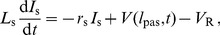
(7)with resistances
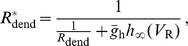


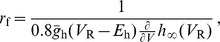


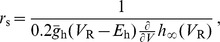
and inductances 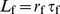 and 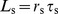.

### Input impedance and transfer impedance of the neuron model

To characterize the voltage response of the linearized model to somatic and dendritic current input we computed the (frequency-dependent) transfer function. For this we expressed the above system in the frequency domain. The cable equation (1) is then written as
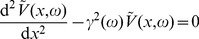
(8)with 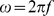 (where frequency 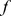 is in Hz) and with propagation constant
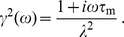



The boundary conditions defined by equations (2) and (3) with an impulse current 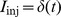 in the distal dendritic segment can be written in the frequency domain as
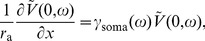
(9)

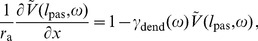
(10)where for the passive soma
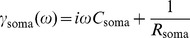
and for the active dendritic segment
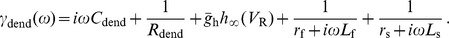



By solving equation (8) with boundary conditions given by equations (9) and (10) we obtained the transfer function of the neuron model:
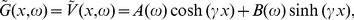
(11)where coefficient 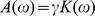 and 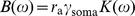, with
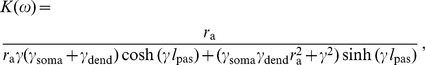
and 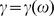, 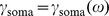, 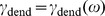.

The absolute value of the (complex-valued) transfer function gives the (frequency-dependent) impedance amplitude of the neuron model. We considered both input impedances (which determine the voltage response at the same location as the input current) and transfer impedances (which determine the voltage response in another location than the input current). To compute the input impedance at the distal dendritic end or the transfer impedance between the active dendritic compartment and the soma, one must let 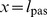 or 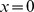 in Equation (11), respectively. When the current input is injected somatically we have 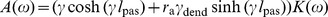 and 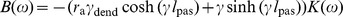 and one can compute the somatic input impedance by letting 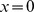. Input and transfer impedances for the neuron with active soma and/or passive distal dendritic segment can be obtained by setting 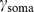 and 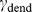 appropriately.

The linear system (Equation (7)) may show a resonant voltage response for particular input frequencies, which is observable in the input impedance and/or transfer impedance as a peak at a non-zero frequency. To describe the quality of the resonance we calculated the so-called “Q-value” (see [31], [32]), which is defined as the ratio of the impedance amplitude at the resonant frequency to the input resistance (i.e. the impedance at zero frequency).

### Numerical computation of impedances of the nonlinear model

We also numerically determined the input impedance and transfer impedance of the nonlinear conductance-based model using the NEURON simulation environment [54]. To compute somatic and dendritic input impedances for Figure 1B,C we injected a so-called ZAP current 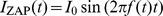, with frequency 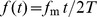, input amplitude 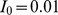 nA, maximum frequency 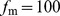 Hz, and stimulus duration 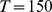 s. At the same location we measured the membrane potential 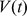 and computed the input impedance as 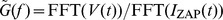, where FFT is the Fast Fourier Transform, an algorithm to efficiently compute the discrete Fourier transform.

To determine the transfer impedances and power spectra for Figure 2D-F, we injected a white noise current (with a duration of 100 s and standard deviation of 0.1 nA) at the distal dendritic end and measured the somatic voltage 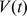. The impedance amplitude profile was determined as 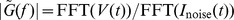. To obtain the results shown in Figure 5, we used the Impedance Tool that is part of NEURON. The time step in the simulations was set to 0.025 ms.

### Model parameters

The neuron models had a uniform leak conductance 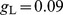 mS/cm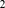 and capacitance 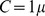
F/cm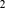 yielding a passive membrane time constant 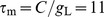 ms. We based the morphological parameters of our standard model (see Figure 1A) on experimental data on cortical pyramidal cell morphologies [25]: length of passive dendritic cable 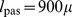
m, length of the active distal dendritic end 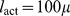
m, dendrite diameter 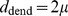
m, surface area of the distal dendritic end 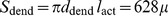
m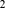, length and diameter of the cylindrical soma 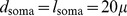
m, surface area of the soma 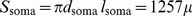
m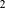. This gave a somatic and dendritic membrane resistance of 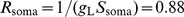 GOhm and 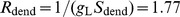 GOhm, respectively, and a somatic and dendritic capacitance of 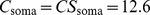 pF and 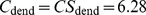 pF. Axial resistivity was set to 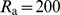 Ohm cm, resulting in an axial resistance 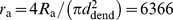 MOhm/cm and a space constant 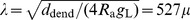
m. The dendritic-soma conductance ratio (which indicates the relative electrical “magnitude” of the two elements and which is inversely proportional to the size of the soma; see [24], [41]) was 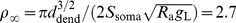, electrotonic length of the passive stretch of dendrite 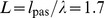. The active dendritic or somatic compartment had 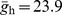 nS. The holding potential was uniformly set to 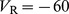 mV. Note that we adjusted the leak reversal potential 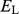 in the active (dendritic or somatic) compartment in order to maintain the same holding potential for the various parameter settings. The parameters of the linearized model (for the default set described above with an active dendritic compartment) were 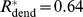 GOhm, 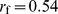 GOhm, 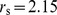 GOhm, 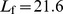 MH, and 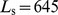 MH.
